# Differences in medical student performance on examinations: exploring score variance between Kolb's Learning Style Inventory classifications

**DOI:** 10.1186/s12909-020-02353-5

**Published:** 2020-11-11

**Authors:** Quentin J. Reynolds, Kurt O. Gilliland, Katie Smith, Joshua A. Walker, Gary L. Beck Dallaghan

**Affiliations:** 1 Department of Psychiatry, Icahn School of Medicine at Mount Sinai, New York, NY USA; 2 Department of Cell Biology and Physiology, University of North Carolina School of Medicine at Chapel Hill, Chapel Hill, NC USA; 3 Office of Medical Education, University of North Carolina School of Medicine at Chapel Hill, Chapel Hill, NC USA; 4 University of North Carolina School of Medicine at Chapel Hill, 108 Taylor Hall, CB 7321, Chapel Hill, NC NC 27599 USA

**Keywords:** Kolb’s theory, Medical students, Learning style inventory, Standardized examinations, Locally-developed examinations

## Abstract

**Background:**

Kolb’s Cycle of Learning Theory acts as a foundational framework for the evolution of knowledge gained by learners throughout their education. Through Kolb’s cycle of experiential learning, one’s preferred way of learning could impact academic achievement in the pre-clinical years of medical education.

**Methods:**

The medical student classes of 2020 and 2021 at a public university in the southeastern U.S. were invited to complete Kolb’s Learning Style Inventory (LSI). For those participants completing the LSI, examination results for their pre-clinical blocks were obtained and matched to the LSI results. Examination scores (locally-developed examinations and customized National Board of Medical Examiners (NBME) final examinations) were compared by LSI classification for each examination using Kruskal-Wallis Test.

**Results:**

Out of 360 possible participants, 314 (87.2%) completed the Learning Style Inventory. Convergers and Assimilators made up 84.1% [Convergers (*n* = 177, 56.4%), Assimilators (*n* = 87, 27.7%)]. Accommodators (*n* = 25, 7.9%) and Divergers (*n* = 25, 7.9%) made up the remaining sample. Accomodators’ scores were significantly lower on locally-developed examinations in Principles of Medicine, Hematology, and Gastrointestinal System. The only NBME examination that demonstrated a significant difference across learning styles was from the Cardiovascular block.

**Conclusions:**

Upon reviewing Kolb’s LSI, our study indicated that performance on the customized NBME examinations minimized the variance in performance compared to locally-developed examinations. The lack of variance across learning styles for all but one NBME final examination appears to provide a more equitable assessment strategy.

## Background

Medical schools are doing more to enhance the diversity of their medical school classes [1]. In doing so, matriculating students hail from a variety of backgrounds, whether it be the type of degree, education level, age, race, and/or ethnicity. Matriculating medical school classes are a kaleidoscope of learners, bringing with them past experiences with various teaching modalities, interests, and positive or negative reinforcement throughout life. It is for these reasons Kolb’s learning styles [2] could be a beneficial way to characterize the diversity of matriculating students without looking into the minute details (i.e., sex, socioeconomic status, race, age, etc.). Kolb developed his theory building off this taxonomy, postulating that knowledge transforms through experiences, initially learning through perceiving the material culminating in processing material [2].

In the 1980s, so-called learning styles were emphasized as a means of matching students’ preferred method of learning to specific teaching modalities [3]. Since then, there is evidence to discount this notion [4–6]. However, upon closer evaluation of Kolb’s Experiential Learning Theory and Learning Style Inventory (LSI), the cycle of learning Kolb developed provides a framework for understanding student learning orientations [7]. This framework could be used to guide approaches to learning medical school content in a way that serves any type of learner.

In Kolb’s model, learning orientations could be described as tensions between active experimentation (AE) vs. reflective observation (RO) and abstract conceptualization (AC) vs. concrete experience (CE). Active experimentation describes a preference for action, contrasting with reflective observation which indicates a propensity to consider possibilities before committing to an action. Abstract conceptualization prefers the development of theories and concepts to explain events whereas concrete experience emphasizes experiential learning [8–10]. From these learning orientations, Kolb established four learning styles: Divergers (concrete experiences with reflection), Assimilators (abstract conceptualization with reflection), Convergers (abstract conceptualization with experimentation), and Accomodators (concrete experiences with experimentation) [6, 11].

The LSI identifies where one enters the cycle of learning. The entry point has been influenced by prior educational experiences and personal preferences. This entry point indicates learner emphasis of action over reflection and abstract thinking over concrete experiences. Therefore, a learning situation may complement a preferred learning style or present a challenge to learning [12].

Regardless of background experiences or learning orientations, medical students must pass multiple-choice examinations during the pre-clinical curriculum. Although our medical school admits a diverse student body, admissions decisions are not based on diverse learning styles. Given our students’ diverse backgrounds and degrees, students with a particular learning style may face challenges adapting to learning medical school content. For this study, we investigated if there were differences based on each examination’s performance across students’ learning style. Previous studies have shown learners who preferred abstract learning (Assimilators and Convergers) perform better on standardized examinations [6, 9, 13]. We hypothesized they would outperform on examinations in our study as well.

## Methods

### Kolb’s Learning Styles Inventory

At a large public school of medicine in the southeastern U.S., all matriculating medical students in the classes of 2020 and 2021 were invited by email to participate and complete the Kolb Learning Style Inventory over a 2-week period at the beginning of the school year. If medical students completed the survey link in the email, they consented to participating in the study. The survey was completed prior to any examinations.

**Fig. 1:**
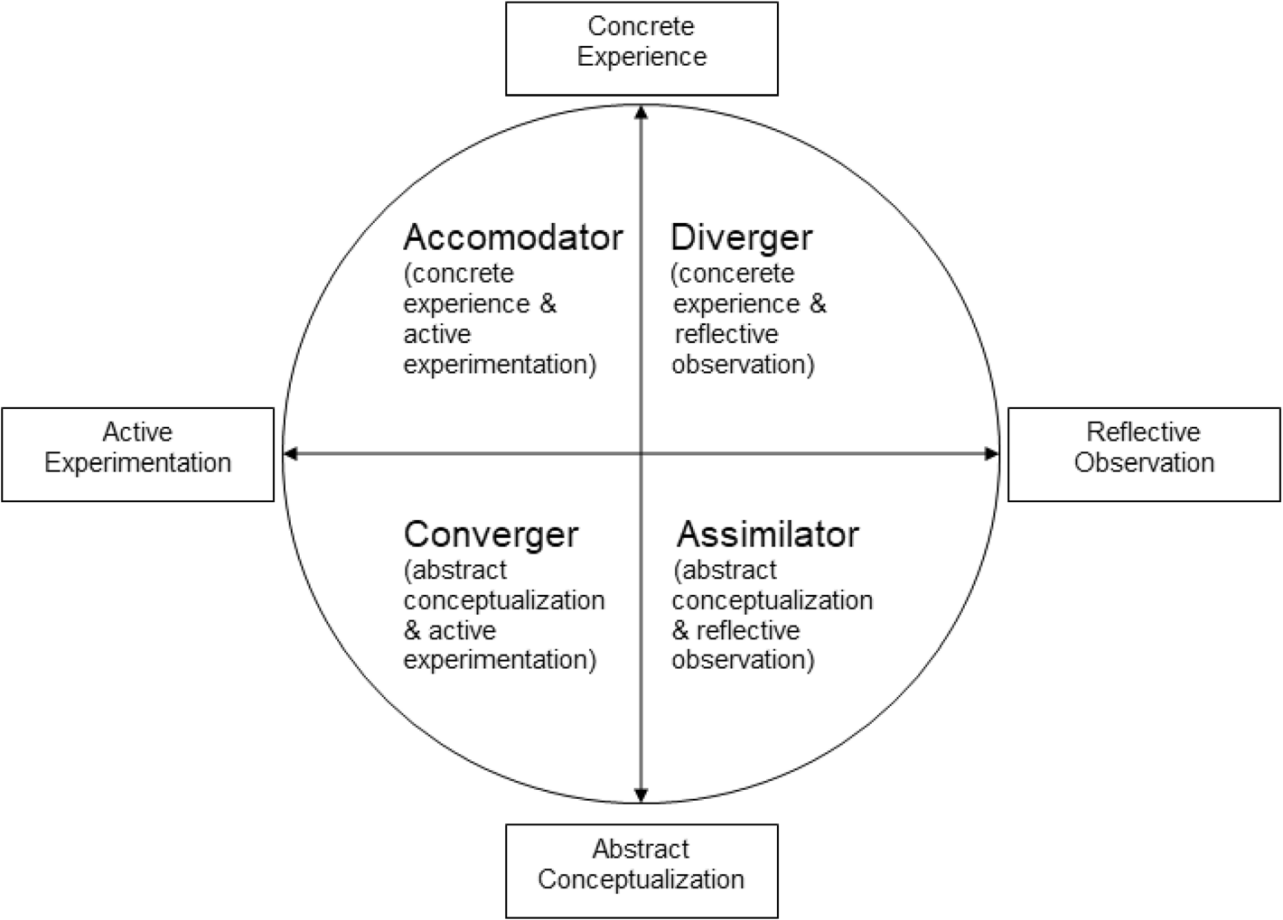
Kolb’s Cycle of Learning. Figure developed based on Kolb’s Cycle of Learning^7^

The newest iteration of the Kolb LSI, version 3.1, was used. It is a 12-item, forced-choice questionnaire ranking participants’ responses against the four learning styles [7]. Participants ranked four statements characterizing their styles for active experimentation versus reflective observation and concrete experience versus abstract conceptualization. Once ranking the forced-choice items, columns are summed to provide a score based on concrete experience, reflective observation, abstract conceptualization, and active experimentation. The original model was built on the idea that learning preferences could be described using two planar dimensions: active experimentation (AE) vs. reflective observation (RO) in the x-dimension, and abstract conceptualization (AC) vs. concrete experience (CE) in the y-dimension. From these two sets of dimensions, Kolb was able to establish four types of learning styles, or preferences. An algorithm was constructed to plot results within the four learning preferences, plotting their learning preference in a RO-AE (x-axis) vs. CE-AC (y-axis) format (Fig. 1). For example, a student may have scores in each area that would map into the Converger quadrant or category.

### Curriculum and assessments

Students undergo 4–8 week organ-system blocks that contain 1–3 examinations throughout the block period. Although blocks differ by numbers of other assignments, a significant portion of their grade is determined on their passing these examinations, and an overall grade of 70% or higher is determined passing. Organ-system course blocks included Principles of Medicine, Immunology, Hematology, Cardiovascular System, Respiratory System, Urinary System, Gastrointestinal System, Endocrinology, Musculoskeletal System, and Reproductive Medicine. The final examination for most blocks is a customized National Board of Medical Examiners (NBME) examination, with the exception of the Principles of Medicine course. All other examinations administered to the medical students were locally-developed examinations. Examination results obtained for this study were all part of the medical students’ normal course of study.

The locally-developed examinations were developed by a cohort of faculty, including but not limited to course directors, guest lecturers, and directors for the pre-clinical phase of the curriculum. This cohort wrote their own multiple-choice questions and answers based on course learning objectives. The NBME examinations were created by a team of block directors as well as directors for the pre-clinical phase of the curriculum. Questions used for the final exam were not comprehensive but assessed new material presented after the most recent examination. These questions were not created by the cohort of faculty; instead, questions were fielded and selected from a larger NBME question bank that matched the course learning objectives. It is important to note that there is no difference in score expectation among all of the examinations, so a passing score of 70% is equitable across all of the exams throughout the pre-clinical phase.

The Office of Medical Education matched examination percentages with LSI results. Identification numbers were replaced with random identifiers. The list was then randomized for anonymity. Data were analyzed using IBM SPSS v. 25 (Chicago, IL). This study was reviewed by the University of North Carolina Institutional Review Board and determined the study was exempt.

### Statistical analysis

LSI categories were compared for each examination. The data violated assumptions for using analysis of variance. Therefore, we used related-samples Wilcoxon Rank Sum Tests and Friedman’s Two-Way Analysis of Variance tests to compare locally-developed examinations. We also used Kruskal-Wallis Tests to compare medical student LSI categories by examination. Post hoc pairwise comparisons were analyzed to determine specific differences between the learning styles for each examination.

## Results

**Fig. 2:**
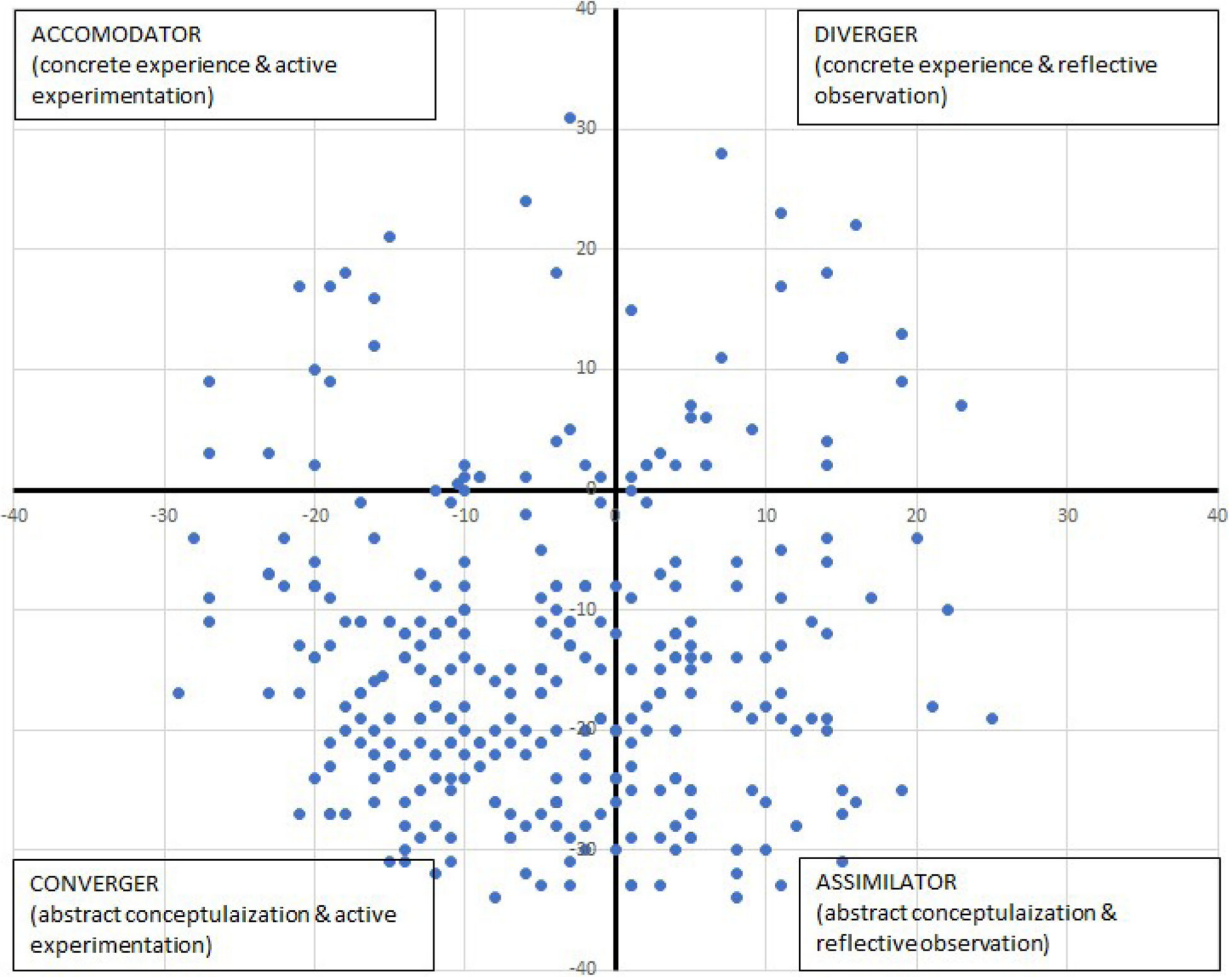
Medical Student Distribution Along Kolb’s Learning Styles Inventory

Out of 360 possible participants, 314 (87.2%) completed the Learning Style Inventory. Convergers and Assimilators made up 84.1% [Convergers (*n* = 177, 56.4%), Assimilators (*n* = 87, 27.7%)]. Accommodators (*n* = 25, 7.9%) and Divergers (*n* = 25, 7.9%) made up the remaining sample. A Cartesian graph depicts the distribution of LSI results (Fig. 2).

Two courses did not offer NBME examinations for their final examination; therefore, we only report the locally developed examination results for those courses (IMM and HEM). Comparisons of performance on examinations by course indicated significant differences for each block, except for Reproductive Medicine (Table 1). The mean percent on the midterm examination was 83.62 versus 83.79 on the NBME final (*p* = .608).

**Table 1:** Comparison of Examination Performance by Learning Style Classification for Two Classes

Examination	Overall Avg Exam Score	Accommodator ***n*** = 25	Assimilator ***n*** = 87	Converger ***n*** = 177	Diverger ***n*** = 25	χ^**2**^	df	P*
Principles of Medicine Mid-term	91.11	88.26	91.09	91.65	91.14	8.37	3	.039*
Principles of Medicine Final	86.14	80.77	87.17	86.59	84.48	13.09	3	.004*
Immunology Exam 1	83.06	80.48	83.81	83.21	81.75	4.63	3	.201
Immunology Exam 2	88.56	85.45	89.16	88.76	87.78	4.99	3	.173
Hematology Exam 1	86.31	81.05	87.12	86.83	84.84	15.51	3	.001*
Hematology Exam 2	89.72	83.81	90.08	90.34	89.79	9.49	3	.023*
Hematology Exam 3	83.04	79.54	83.83	83.24	81.78	5.04	3	.169
Cardiovascular Exam 1	84.45	80.03	85.24	84.81	83.63	5.96	3	.114
Cardiovascular Exam 2	82.91	78.79	82.75	83.94	79.87	11.35	3	.010*
Cardiovascular NBME	83.00	79.38	82.28	83.90	82.32	9.97	3	.019*
Respiratory Exam 1	88.66	86.50	88.58	89.22	87.12	6.87	3	.076
Respiratory Exam 2	86.28	80.45	87.11	85.38	85.83	7.33	3	.062
Respiratory NBME	85.75	85.44	84.52	86.51	84.52	4.52	3	.211
Urinary Exam 1	82.61	79.28	82.48	82.98	83.28	2.89	3	.409
Urinary NBME	81.61	77.97	81.77	81.86	82.55	6.28	3	.099
GI Mid-term	82.61	77.87	83.44	82.95	81.71	12.10	3	.007*
GI NBME	81.77	79.09	81.73	82.26	80.97	3.72	3	.294
Musculoskeletal Exam 1	92.39	89.26	92.66	92.74	92.00	5.81	3	.121
Musculoskeletal Exam 2	92.46	89.57	93.07	92.69	91.63	6.74	3	.081
Musculoskeletal NBME	82.82	84.92	86.31	85.85	84.67	3.76	3	.289
Endocrinology Mid-term	84.73	83.12	85.15	84.97	82.72	3.79	3	.285
Endocrinology NBME	87.88	87.96	86.85	88.40	87.76	1.64	3	.651
Reproductive Medicine Mid-term	83.63	83.66	83.51	83.81	82.39	1.06	3	.787
Reproductive Medicine NBME	83.79	83.24	82.63	84.76	81.60	3.83	3	.280

*Differences significant when *p* < .05

This table details the average score for each examination for each learning style as well as the overall for the entire sample studied

Significant differences were found with locally-developed examinations in Principles of Medicine, Hematology, and Gastrointestinal System. Both examinations in Principles of Medicine were significantly different (Mid-term: χ^2^(3) = 8.37, *p* = .039; Final: χ^2^(3) = 13.09, *p* = .004). Post hoc tests for the midterm identified significant differences between Convergers (μ = 91.65) and Accommodators (μ = 86.59) (U = − 51.21, *p* = .008). For the final, Convergers (μ = 86.59; U = − 64.33, *p* = .001) and Assimilators (μ = 87.17; U = − 69.57, *p* = .001) scored higher than Accommodators (μ = 80.77).

Similar results were found for the first Hematology examination (χ^2^(3) = 15.51, *p* = .001). Post hoc tests indicated Convergers (μ = 86.83; U = − 70.93, *p* = .002) and Assimilators (μ = 87.12; U = − 65.46, *p* = .002) scored higher than Accommodators (μ = 81.05). Significant differences were also identified on the second Hematology examination (χ^2^(3) = 9.49, *p* = .023). Post hoc tests demonstrated significant differences between Convergers (μ = 90.34; U = − 55.12, *p* = .004), Assimilators (μ = 90.08; U = − 55.67, *p* = .007), and Divergers (μ = 89.79; U = − 69.38, *p* = .007) with Accommodators (μ = 83.81).

Finally, the Gastrointestinal System midterm was also significantly different (χ^2^(3) = 12.10, *p* = .007). Post hoc tests indicated Convergers (μ = 82.95; U = − 62.36, *p* = .001) and Assimilators (μ = 83.44; U = − 63.05, *p* = .002) scored higher than Accommodators (μ = 77.87). It should be noted that the Cardiovascular System examination #2 appeared statistically significant (χ^2^(3) = 11.35, *p* = .010); however, inspection of post hoc tests using the Bonferroni correction for multiple tests did not find significant differences.

The only NBME final that was significantly different was in the Cardiovascular System block (χ^2^(3) = 9.97, *p* = .019). Post hoc tests indicated Convergers (μ = 83.90; U = − 51.68, *p* = .008) scored higher than Accommodators (μ = 79.38).

## Discussion

Based on our study findings, the use of locally-developed examinations may be poor indicators for proficiency in any subject. This conclusion was drawn based on the significant score differences in which Accommodators had worse outcomes, and Convergers and Assimilators consistently scored higher than their peers. What is most exceptional is that when compared to NBMEs, this statistical significance and variance in scores were minimized across learning styles. The only NBME examination in which this was not the case is the Cardiovascular System block, but the significance in scores does not resemble the typical dynamic as mentioned above and in previous research [6].

Variations in locally developed examination performance may be explained in part by the learning styles. According to Kolb, Accommodators have a preference for concrete examples [7]. As noted by An and Carr [14], needing concrete examples may be associated with novice learners, who most likely have not learned how to abstract general rules in their learning process. Consequently, test-expectancy effects may explain why Accommodators can perform better on NBME examinations, because there are many resources available that give them concrete experiences with multiple-choice examinations of that nature [15]. They therefore have a better sense of what to expect on an NBME examination versus a locally-developed examination.

Additionally, our findings that most NBME examination scores were lower than locally-developed examinations contradict findings from other studies [9, 16]. The lack of statistical significance and paired comparisons implies that customized NBME examinations may better reflect student performance than locally-developed examinations. Given the diversity of students being accepted to medical school who may be at varying points in Kolb’s cycle of learning, administering the NBME examinations for all summative assessments may be warranted for fairness, whether it be the midterm or final.

We have hypothesized the difference in the Cardiovascular System NBME examination may be the result of the customization of the examination. The faculty for this block evaluate the item analysis from the previous year’s examination to remove items that were easy (i.e., with high percent correct values) or flawed (i.e., with a low discrimination indices). We theorize that this distinct type of customization, which was not used in any of the other NBME exams, may have resulted in the examination mirroring locally-developed examinations. As we have shown, the locally-developed examinations favor Convergers and Assimilators.

Locally-developed examinations are created by the block director(s) themselves, and there is research that supports that the development of the curriculum for any given block can be skewed towards a specific preference or group of preferences [17]. Prior research indicated medical professionals are predominantly Convergers and Assimilators, which may influence how they construct examination items [17]. For concrete thinkers, variations in how individual faculty write examinations may pose challenges for encoding strategies to do well on these examinations [15]. Given sufficient training, locally-developed examinations may reflect the difficulty found in NBME-style questions [18, 19]. Training faculty not only to write but also to peer-evaluate questions could offer more equanimity in assessments [20].

It has also been shown that learning style classifications evolve over time [8]. For these studies we administered the LSI at only one point. For future studies, it may be interesting to administer the LSI at multiple points in time to determine if there is a shift toward being more of a Converger or Assimilator as a result of the medical school environment [8]. This same study could then be continued into the clinical training environment since learning in that setting is much different than traditional classroom learning. The types of assessments employed in the clinical settings may also be influenced by LSI results, which has not really been studied to date.

Customized NBME examinations have been available for many years; the time commitment and cost for the examinations have been a deterrent for full execution in the pre-clinical curriculum. Although we have opted to utilize them entirely within the pre-clinical curriculum, not every medical school will have the resources to do that.

This study is limited in that it was conducted at a single institution that resulted in groupings that had no more than 25 students in two of the LSI groups. However, our sample compares to other studies with similar breakdowns in Kolb’s classifications for medical professionals [17]. Further study is warranted to determine if this dip in nationally standardized examinations was unique to these classes. Item analyses for the customized examinations should also be further explored to determine if they represent competence versus discrimination of performance.

## Conclusions

By identifying the learning styles of matriculating medical students, our study indicates that use of a nationally-standardized examination may minimize variance in scores across learning styles. It is important to note that the variance in NBMEs was insignificant for all examinations except for the Cardiovascular NBME. Therefore, it would be understood that the remaining NBMEs show no discrimination by learning presentation versus learning processing. One way to help concrete thinkers may be to provide practice questions from locally developed examinations to help them better prepare.

## Data Availability

Data is available upon request from corresponding author.

## Abbreviations

AE: Active experimentation
RO: Reflective observation
AC: Abstract conceptualization
CE: Concrete experience (CE)
NBME: National Board of Medical Examiners
LSI: Learning Styles Inventory
